# Arthroscopic physeal-sparing TFCC foveal repair via a single extracapsular transverse bone tunnel in adolescents: a retrospective cohort study

**DOI:** 10.1186/s12891-025-09246-y

**Published:** 2025-10-24

**Authors:** Zeming Lei, Xinzhu Wang, Liangzi Yin, Hui Zhang, Yang Yao, Yansheng Wang

**Affiliations:** 1 Hand Surgery Ward 5, Central Hospital Affiliated to Shenyang Medical College, No. 5, Nanqi West Road, Tiexi District, Shenyang, Liaoning Province 110000 China; 2 Department of Orthopedic Surgery, Shengjing Hospital of China Medical University, No. 36, Sanhao Street, Heping District, Shenyang, Liaoning Province 110000 China; 3 Orthopedics Department Ward 1, Jin Qiu Hospital of Liaoning Province (Geriatric Hospital of Liaoning Province), No.317 Xiaonan Street, Shenhe District, Shenyang, Liaoning Province 110000 China; 4 Laboratory for Hand Bone and Joint Disease, Shenyang Institute of Hand Surgery, No. 5, Nanqi West Road, Tiexi District, Shenyang, Liaoning Province 110000 China

**Keywords:** Triangular fibrocartilage complex, Arthroscopic repair, Foveal tear, Transosseous suture, Adolescent injury

## Abstract

**Background:**

Conventional arthroscopic techniques for triangular fibrocartilage complex (TFCC) foveal repair risk iatrogenic physeal injury in adolescents due to longitudinal oblique bone tunnels traversing the distal ulnar physis. This study evaluates a novel physeal-sparing technique utilizing a single extracapsular transverse bone tunnel to avoid growth plate compromise.

**Methods:**

A retrospective cohort study included 13 adolescents (10 males, 3 females; median age 15.0 years, range 13–17) with Palmer 1B TFCC foveal tears confirmed by MRI and arthroscopy. All patients underwent arthroscopic transosseous repair through a horizontal transverse bone tunnel drilled proximal to the physis at the ulnar neck level under fluoroscopic guidance. Outcomes included pain scores (visual analogue scale [VAS]), grip strength ratio (affected/unaffected side), wrist range of motion (ROM), distal radioulnar joint (DRUJ) stability, and functional scores: the Modified Mayo Wrist Score (MMWS) and Disabilities of the Arm, Shoulder, and Hand (DASH) score. Mean follow-up was 29.0 months (range 12–60).

**Results:**

At a mean follow-up of 29.0 months, the median VAS score decreased from 5.0 to 1.0 postoperatively. The mean grip strength ratio improved from 91.3% to 102.9%. Median wrist flexion-extension ROM increased from 130.0° to 134.0°, and median pronation-supination ROM improved from 150.0° to 170.0°. The median MMWS improved from 75.0 to 95.0, and the median DASH score decreased from 31.7 to 4.2. No clinical or radiographic signs of physeal injury (e.g., ulnar shortening, angular deformity) or DRUJ instability were observed.

**Conclusions:**

This fluoroscopically guided physeal-sparing technique achieved anatomical TFCC restoration and functional recovery in adolescents by avoiding the physis through an extracapsular transverse tunnel. The absence of growth-related complications supports its safety, though long-term studies are warranted.

## Introduction

 The triangular fibrocartilage complex (TFCC) is a critical stabilizer of the distal radioulnar joint (DRUJ), with its proximal component (pc-TFCC) anchoring to the ulnar fovea to maintain joint kinematics [1]. In adolescents, TFCC injuries are increasingly prevalent due to rising participation in competitive sports and high-energy activities [2]. Notably, the incidence of wrist sports injuries in this population surpasses that of adults [3], yet the majority of surgical techniques and outcome studies remain focused on adults, leaving a significant gap in pediatric-specific management strategies [4, 5].

Among adolescents, Palmer 1B foveal tears (Atzei classes 2–3) are the most common TFCC injuries requiring surgical intervention [4]. These injuries disrupt the pc-TFCC’s foveal attachment, leading to DRUJ instability, chronic ulnar-sided wrist pain, and functional impairment [6, 7]. Anatomical reattachment of the pc-TFCC to the fovea is essential to restore stability. Transosseous suture repair is increasingly preferred over capsular repairs or suture anchors due to its biomechanical superiority in achieving secure foveal fixation [8, 9]. However, conventional transosseous techniques rely on longitudinal oblique bone tunnels [10, 11] that traverse the distal ulnar physis in skeletally immature patients, posing a substantial risk of iatrogenic growth arrest or angular deformity [12, 13]. This limitation is particularly critical in adolescents, where the physis remains active and vulnerable to iatrogenic physeal injury [14].

Recent advances in pediatric orthopedic surgery emphasize physeal-sparing approaches to preserve growth potential, as exemplified in anterior cruciate ligament (ACL) reconstruction [15]. Similarly, in TFCC repair, modifying tunnel trajectory to avoid the physis could mitigate growth plate injury while maintaining biomechanical efficacy. Existing pediatric TFCC studies report promising outcomes with transosseous techniques [11, 16], yet none address the inherent physeal risks of longitudinal tunnels.

In this study, we introduce a novel arthroscopic physeal-sparing technique for TFCC foveal repair in adolescents. By replacing longitudinal oblique tunnels with a single extracapsular transverse bone tunnel drilled at the ulnar neck level under fluoroscopic guidance, we aim to (1) achieve anatomical pc-TFCC reattachment, (2) eliminate physeal violation, and (3) validate functional outcomes and safety in a pediatric cohort.

## Methods

### Study design and patient selection

This retrospective cohort study (a small case series) was conducted following Institutional Review Board (IRB) approval from the Central Hospital Affiliated to Shenyang Medical College (KE-2024-001(02)). A total of 13 adolescent patients (10 males, 3 females; median age, 15.0 years; range, 13–17 years) diagnosed with Palmer 1B TFCC foveal tears (Atzei class 2 or 3) underwent arthroscopic repair between July 2019 and December 2023.

Inclusion criteria were as follows: age ≤ 19 years at the time of surgery, open distal ulnar physis on radiographic assessment, symptomatic DRUJ instability confirmed by a positive ballottement test (a manual stress exam detecting excessive ulna movement relative to the radius) after failing six weeks of conservative management (protective splinting), and a foveal TFCC avulsion confirmed via preoperative magnetic resonance imaging (MRI) and arthroscopic examination with a positive hook test. Patients were excluded if they had concomitant fractures of the carpus, distal radius, or ulna, a follow-up duration of less than 12 months, Atzei class 4 or 5 TFCC injuries, or ulnar impaction syndrome. All procedures were performed by a senior hand surgeon with Level 4 expertise [17].

### Surgical technique

#### Preoperative assessment and patient positioning

Preoperative evaluation included a comprehensive physical examination, DRUJ ballottement testing, and imaging studies (standard wrist radiographs and MRI). Under brachial plexus block anesthesia and tourniquet control, the patient was positioned supine with the operative arm placed in a traction tower. The wrist was distracted with approximately 10 lb of longitudinal traction. Standard 3–4 and 6R arthroscopic portals were established for visualization and instrumentation.

#### Arthroscopic examination and TFCC Preparation

After synovectomy and debridement of synovial hyperplasia, TFCC foveal avulsion was confirmed via a hook test. The ulnar fovea was debrided and decorticated to optimize healing.

#### Physeal-sparing transverse bone tunnel creation and transosseous suture fixation

**Fig. 1:**
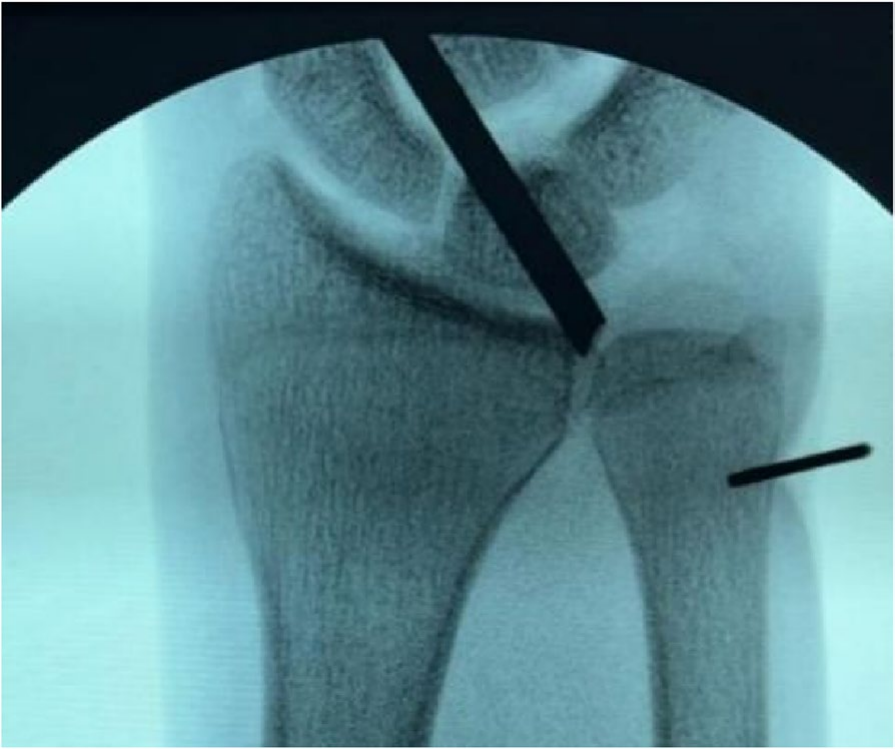
Under fluoroscopic guidance, a single 1.2-mm horizontal transosseous tunnel was drilled at the ulnar neck level, proximal to the physis and directly beneath the fovea to ensure complete avoidance of the physis and prevent growth disturbance. The X-ray image shows the positioning of the transosseous tunnel at the ulnar neck level

A 2-cm longitudinal incision was made along the ulnar border of the wrist, proximal to the ulnar styloid. Under fluoroscopic guidance, a single horizontal transosseous tunnel (1.2 mm in diameter) was drilled at the ulnar neck level, proximal to the physis and directly beneath the fovea, ensuring complete avoidance of the physis to prevent growth disturbance (Fig. 1, suggested schematic of bone tunnel location).

**Fig. 2:**
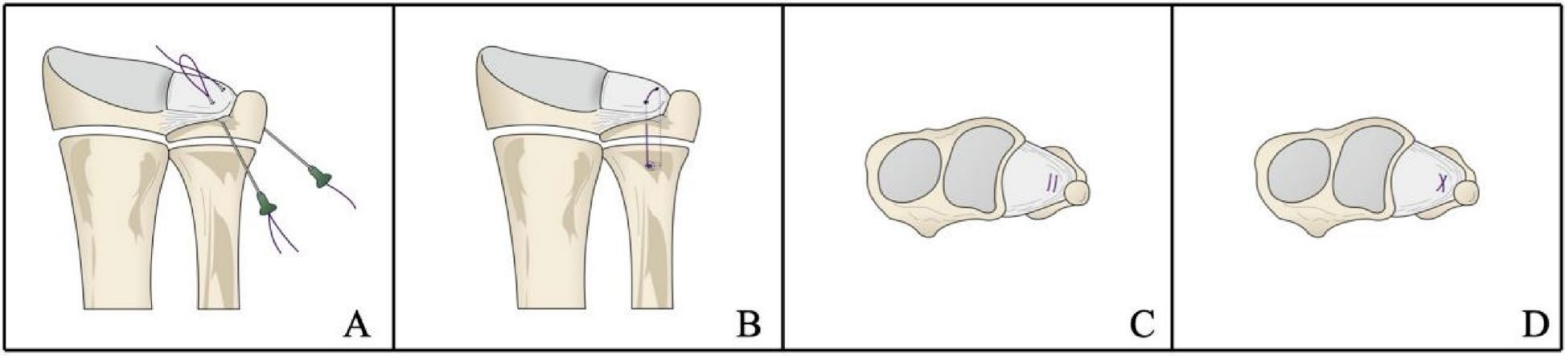
Schematic representation of the transosseous suture configurations used in the physeal-sparing TFCC foveal repair. **A** A single U-type suture configuration is initially created by passing the sutures through the TFCC and retrieving them externally. **B** When both ends of the suture are tightened, the TFCC is compressed against the fovea under arthroscopic visualization. **C** To increase the contact area and create a surface contact, an additional suture is placed in the same manner, forming a double U-type configuration. **D** Alternatively, when combined with the first suture, the configuration can form an X-type pattern

A suture lasso was prepared using a 2 − 0 PDS suture through a 21G needle. The first suture was placed dorsally, passing beneath the ulnar styloid base and penetrating the dorsal margin of the TFCC under arthroscopic guidance. A second suture was placed volarly, passing beneath the ulnar styloid base and penetrating the volar margin of the TFCC. Both sutures were retrieved through the 6R portal and looped externally. Then, the loop was pulled out from the ulnar incision. When both ends of the suture were tightened, the suture formed a single U-type configuration, pressing the TFCC against the foveal region under arthroscopic visualization. To increase the contact area and create a “surface” contact, an additional suture was applied in the same manner, forming a double U-type suture or, when combined with the first suture, an X-type suture configuration (Fig. 2, suggested schematic of suture configuration). This ensured better compression of the TFCC against the fovea.

Once the repair was completed, the dorsal sutures were then passed through the transverse bone tunnel to the volar side. With the wrist in a neutral rotation and the DRUJ reduced, the sutures were tied securely. The hook test and DRUJ stability were reassessed to confirm proper fixation. Finally, the surgical site was irrigated, and the incision was closed in layers.

#### Postoperative rehabilitation protocol

Postoperatively, the wrist and forearm were immobilized in a long arm cast with the elbow flexed at 90° and the forearm in 45° supination for three weeks. From weeks 3 to 6, the cast was transitioned to a short arm splint, allowing controlled wrist flexion-extension and mid-range forearm rotation. After the splint was removed, patients progressively regained full pronation-supination and wrist flexion-extension and began gradual weight-bearing exercises. By three months postoperatively, most patients achieved full forearm rotation and wrist motion. Return to unrestricted daily activities was allowed at 4–6 months, while strenuous sports were permitted no earlier than 6–8 months postoperatively.

### Outcome measures and data collection

Clinical outcomes were assessed preoperatively and at the final follow-up (mean, 29.0 months; range, 12–60 months). Pain levels were evaluated using the VAS, grip strength was measured as the affected/unaffected side ratio, and wrist ROM was assessed for flexion-extension and pronation-supination. DRUJ stability was confirmed using the ballottement test. Patient-reported outcomes included the Modified Mayo Wrist Score (MMWS) and Disabilities of the Arm, Shoulder, and Hand (DASH) score. Any postoperative complications, including infection, ulnar nerve injury, and iatrogenic fracture, were documented through clinical evaluation, while potential physeal injury, such as ulnar shortening or angular deformity, was assessed using standard radiographic follow-up.

Any postoperative complications, including infection, ulnar nerve injury, iatrogenic fracture, or physeal injury, were documented through clinical and radiographic assessment as appropriate.

### Statistical analysis

Statistical analyses were performed using SPSS 27 (IBM, Armonk, NY, USA). Paired-sample t-tests were employed to compare the differences in variables before and after surgery. The normality of the paired differences (i.e., post – pre values) was assessed using the Shapiro–Wilk test, and all difference scores were found to conform to a normal distribution (*p* > 0.05). According to standard statistical guidance, the paired t-test requires normality of the difference scores rather than the individual distributions. Therefore, the use of paired t-tests in this study is appropriate. Variables with normal distribution are presented as mean ± standard deviation (SD), while non-normally distributed data are expressed as median (Q1, Q3). Categorical variables are summarized as frequency (%). A significance level of *p* < 0.05 was used.

## Results

A total of 13 patients underwent the modified arthroscopic physeal-sparing transosseous repair for TFCC foveal avulsion between July 2019 and December 2023, with no loss to follow-up (Table 1). The median age at the time of injury was 15.0 years (range, 13–17 years). There were 10 males and 3 females, with the right wrist affected in 9 cases (69.2%) and the left wrist in 4 cases (30.8%). The primary injury mechanism was fall-related trauma in 9 patients (69.2%), while the remaining 4 patients (30.8%) sustained wrist sprains. The mean time interval from injury to surgery was 15.0 weeks (range, 6–25 weeks).

**Table 1:** Patients demographic and clinical characteristics (*n* = 13)

Characteristics	Value
Age (years)	15.0 (13.5, 17.0)
Sex	
Female	3 (23.1%)
Male	10 (76.9%)
Injured wrist	
Left	4 (30.8%)
Right	9 (69.2%)
Trauma mechanism	
Fall	9 (69.2%)
Wrist sprain	4 (30.8%)
Injury to surgery (weeks)	15.0 ± 7.1
Follow-up (months)	29.0 ± 13.6
Atzei-EWAS classification	
Class 2	7 (53.9%)
Class 3	6 (46.2%)

Continuous variables are expressed as mean ± SD or as median (Q1, Q3), as appropriate. Categorical variables are presented as number and percentage

During arthroscopy, all 13 patients demonstrated positive hook test results, confirming TFCC foveal avulsion. According to the Atzei classification, 7 patients (53.9%) had class 2 injuries, and 6 patients (46.2%) had class 3 injuries. No concomitant interosseous ligament or carpal ligament injuries were identified. The mean follow-up duration was 29.0 months (range, 12–60 months).

No postoperative complications, including wound infection, ulnar nerve injury, or iatrogenic fracture of the ulnar styloid, were observed. Standard radiographic follow-up revealed no apparent physeal injury, such as ulnar shortening or angular deformity. At each follow-up, all patients tested negative for the ballottement test, with no residual DRUJ instability detected.

At final follow-up, all functional and patient-reported outcomes significantly improved compared to preoperative values (Table 2). The median VAS score for wrist pain significantly decreased from 5.0 preoperatively to 1.0 postoperatively (*p* < 0.01). Compared to the contralateral uninjured side, the mean grip strength ratio improved from 91.3% to 102.9% (*p* < 0.01). Median wrist flexion-extension ROM increased from 130.0° to 134.0° (*p* < 0.05), while median pronation-supination ROM improved from 150.0° to 170.0° (*p* < 0.01). The median MMWS increased from 75.0 preoperatively to 95.0 postoperatively (*p* < 0.01). The median DASH score decreased from 31.7 to 4.2 (*p* < 0.01), indicating substantial functional recovery. According to the MMWS grading, 10 patients achieved excellent outcomes, and 3 patients had good outcomes at final follow-up.

**Table 2:** Assessments before operation and at final follow-up

Assessments	Preoperative	Postoperative	Postoperative-Preoperative Difference	*p* value
VAS	5.0 (3.5,6.5)	1.0 (0.0,1.0)	− 4.5 ± 1.9	*p <* 0.01
Grip strength ratio	91.3%±16.7%	102.9%±12.5%	11.6%±6.2%	*p <* 0.01
Wrist ROM				
Flexion-extension(°)	130.0 (102.0,140.0)	134.0 (126.0,139.0)	8.1 ± 10.9	*p <* 0.05
Pronation–supination(°)	150.0 (138.0,157.5)	170.0 (162.5,172.5)	22.4 ± 18.1	*p <* 0.01
MMWS	75.0 (57.5,80.0)	95.0 (92.5,100.0)	25.0 ± 15.3	*p <* 0.01
DASH score	31.7 (24.6,37.5)	4.2 (3.3, 5.4)	−27.0 ± 7.8	*p <* 0.01

Continuous variables are expressed as mean ± SD or as median (Q1, Q3), as appropriate

## Discussion

The results of this small case series demonstrated satisfactory clinical outcomes in adolescent patients who underwent arthroscopic physeal-sparing transosseous foveal repair of the TFCC using a single extracapsular transverse bone tunnel.

Foveal avulsion of the proximal component of the TFCC (pc-TFCC) (Atzei class 2 or 3 tears) disrupts the primary stabilizing structure of the DRUJ and requires anatomical reattachment to restore joint stability [1]. While various repair techniques have been reported, transosseous suture repair and suture anchor repair are considered the most effective methods for foveal fixation [18]. Nakamura et al. [10] first described an arthroscopic transosseous tunnel technique for TFCC foveal repair using two longitudinal oblique bone tunnels. This technique was later refined by Shinohara et al. [11], who improved tunnel placement under direct arthroscopic visualization of the DRUJ. Since then, various modified surgical techniques have been published. Jegal et al. [19] introduced a dual-bone tunnel technique to anatomically repair both the dc-TFCC and pc-TFCC. In this technique, a syringe needle with one suture was inserted into the fovea, passing through each tunnel separately. The sutures were then pulled out from the distal ulnar styloid incision and tied down over the joint capsule, achieving anatomical reattachment of the TFCC components. Liu et al. [20, 21] further refined this approach by employing a ligament-specific transosseous technique to separately restore the deep dorsal and volar limbs of the TFCC, achieving near-anatomical reconstruction with promising clinical outcomes.

Although these dual-bone tunnel techniques have been widely adopted in adults, their application in children and adolescent populations remains limited. One major concern is that the traditional longitudinally oblique tunnels pass through the distal ulnar physis, potentially increasing the risk of physeal arrest, ulnar shortening, or angular deformity. Pfanner et al. [22] emphasized the importance of preventing physeal injury in pediatric orthopedic procedures by avoiding hardware placement across the growth plate. Although transosseous suture repair theoretically minimizes implant-related complications, even small-diameter K-wires used for tunnel creation can disrupt the physis, as demonstrated in experimental models [13]. Tang et al. [12] reported that using 1.6-mm K-wires to fix adolescent triplane distal tibia fractures led to premature physeal closure in some cases, underscoring the potential risks associated with transphyseal drilling.

To mitigate this risk, our study introduces a physeal-sparing technique by modifying the conventional longitudinal oblique bone tunnels into a single extracapsular transverse tunnel located proximal to the distal ulnar physis and directly beneath the fovea. This approach effectively avoids physeal violation while preserving the advantages of transosseous suture repair. Additionally, placing the tunnel extracapsularly reduces intra-articular trauma and minimizes interference with joint mechanics. Compared to longitudinal oblique dual-bone tunnel techniques, our method may also lower the risk of iatrogenic ulnar styloid fracture by reducing cortical weakening at the ulnar styloid base.

Another key feature of our technique is its emphasis on achieving a broader contact area between the TFCC and the fovea, which may enhance healing. The traditional dual-bone tunnel method provides a line contact, while suture anchor fixation offers a point contact [10, 23, 24]. However, anatomical studies have shown that the native TFCC insertion is not a single point or line but rather a broad surface area measuring approximately 29.67 mm² [25]. Experimental studies suggest that ligament-to-bone healing is improved when the contact area is increased [26]. In addition, compared to single-line suturing, double-line suturing can reduce the risk of TFCC cut-through and enhance repair stability by distributing tension and increasing the contact area between the TFCC and the fovea [27, 28]. Our technique involves inserting the ulnar margin of the TFCC with two needles positioned next to the ulna, without passing through a fixed bone tunnel. Because there is no limitation imposed by a pre-drilled bone tunnel, the range of insertion angle adjustment is larger, making it easier and more precise to suture the ulnar edge of the TFCC.

Our findings align with previous reports on pediatric TFCC repair. Shinohara et al. [11] assessed 11 patients, including four adolescents (aged 15–18 years), who underwent two-tunnel transosseous TFCC foveal repair. Their results demonstrated significant improvements in grip strength and functional scores, with complete pain resolution in three patients and only mild residual pain in one case. Trehan et al. [16] reviewed 43 children and adolescent patients with TFCC injuries, reporting favorable outcomes following transosseous repair. However, both studies utilized longitudinally oblique transosseous tunnels, raising concerns about potential physeal injury. Our study expands on these findings by demonstrating that a transverse extracapsular bone tunnel can achieve similar clinical outcomes while reducing physeal risk.

One controversial aspect of TFCC repair is whether the distal component of the TFCC (dc-TFCC) should also be addressed in cases of Atzei class 2 injuries. The pc-TFCC, which inserts into the fovea, plays a dominant role in DRUJ stability, whereas the dc-TFCC primarily supports the ulnar carpus [23, 29]. Biomechanical studies suggest that isolated pc-TFCC disruption leads to significant DRUJ instability, whereas isolated dc-TFCC injury does not [30]. Nam et al. [31] reported that patients with combined pc-TFCC injury and ulnar styloid nonunion achieved comparable outcomes to those with isolated pc-TFCC injuries when only the foveal component was repaired. Several clinical studies have demonstrated that reconstructing the pc-TFCC alone effectively restores wrist function and relieves pain, even in the presence of concurrent dc-TFCC tears [21, 24, 32]. Given that the dc-TFCC has a relatively rich blood supply, it may undergo spontaneous healing once DRUJ stability is restored [33, 34]. Based on these findings, our approach focused solely on foveal reattachment, which resulted in excellent or good functional outcomes in all patients.

This study has several limitations. First, it is a retrospective analysis with a relatively small sample size, though comparable to previous pediatric TFCC studies [22, 35, 36]. Second, the follow-up period is limited, and longer-term studies are needed to assess the durability of our technique. Third, this study did not directly compare clinical outcomes between the transverse extracapsular tunnel technique and traditional longitudinal oblique transosseous techniques, which would provide stronger evidence for its advantages. Finally, the biomechanical properties of our modified technique have not been validated through cadaveric or computational modeling studies. Future research should focus on quantifying the biomechanical stability and healing characteristics of different transosseous configurations.

## Conclusions

Our small case series suggests that arthroscopic physeal-sparing TFCC foveal repair using a single extracapsular transverse bone tunnel is a safe and effective approach for adolescent patients. By avoiding the physis and optimizing TFCC-fovea contact, this technique provides stable DRUJ restoration while minimizing the risk of growth-related complications. These findings support the safety and functional efficacy of the technique, though larger and longer-term studies are needed to confirm these results.

## Data Availability

The datasets used and/or analysed during the current study are available from the corresponding author on reasonable request.

## Abbreviations

TFCC: Triangular fibrocartilage complex
ROM: Range of motion
DRUJ: Distal radioulnar joint
ACL: Anterior cruciate ligament
IRB: Institutional Review Board
VAS: Visual analogue scale
MMWS: Modified Mayo Wrist Score
DASH: Disabilities of the Arm, Shoulder, and Hand
dc-TFCC: Distal component of the TFCC
pc-TFCC: Proximal component of the TFCC
